# Miscellany

**Published:** 1908-05

**Authors:** 


### MISCELLANY

Mr. W. Sheldon Bull, of Buffalo, contributed an article to the April number of *Good Housekeeping* in which he relates his experience with the backyard goat dairy, that he has established on his premises. Elsewhere in this magazine we reprint Mr. Bull's paper and commend it to those interested, especially to those who are compelled to raise babies through artificial feeding. Goat's milk is said to be next to mother's milk in nutritive elements; causing little or no digestive disturbance. In Italy, and some other countries, infants and children nurse the goat direct, without inconvenience to their digestive functions. Mr. Bull can supply the milk to a limited extent.

Battle and Company, of Saint Louis, have issued pamphlet No. 5, containing a plate showing subcoracoid dislocation of shoulder. This is one of a series of eighteen illustrations of dislocations issued as a complement to long bone fractures.

Dr. L. L. Seaman, of New York, offers a prize of $100.00 for the best essay on The Economic Waste Due to Occupational Diseases. The time limit for the presentation of the essays, first fixed at April 1, has been extended to June 15, 1908. The essay should contain not more than 5,000 words, must be signed by a pen name and the real name and address sent in a sealed envelope to the director of the Museum of Security of the American Institute of Social Service, 231-241 West 39th Street, New York.

The State Civil Service Commission will hold examinations on May 9, 1908, for among others the following positions: Assistant Civil Engineer, $5.00 to $6.00 a day; Bookkeeper, fourth grade, $720.00; Health Officer, Town of Brasher, St. Lawrence County; Physician, State Hospitals and Institutions, $900.00 and maintenance; Stereotyper, State School for the Blind, $600.00 and maintenance; Telephone Operator, State and County Offices and Institutions, $360.00 to $720.00; Woman Officer, State Institutions, $300.00 to $360.00 and maintenance. The last day for filing applications for these positions is May 2.

Full information and application forms for any of these examinations may be obtained by addressing the Chief Examiner of the Commission, Charles S. Fowler, Albany.

